# Risk taking of educated nematodes

**DOI:** 10.1371/journal.pone.0205804

**Published:** 2018-10-25

**Authors:** Denis S. Willett, Hans T. Alborn, Lukasz L. Stelinski, David I. Shapiro-Ilan

**Affiliations:** 1 Cornell University, Entomology Department, Cornell AgriTech, Geneva, NY, United States of America; 2 Agricultural Research Service, United States Department of Agriculture, Center for Medical, Agricultural and Veterinary Entomology, Gainesville, FL, United States of America; 3 University of Florida, Entomology and Nematology Department, Citrus Research and Education Center, Lake Alfred, FL, United States of America; 4 Agricultural Research Service, United States Department of Agriculture, SE Fruit and Tree Nut Research Laboratory, Byron, GA, United States of America; George Washington University School of Medicine and Health Sciences, UNITED STATES

## Abstract

Nematode parasites rely on successful host infection to perpetuate their species. Infection by individual nematode parasites can be risky, however; any one individual could be killed by the host’s immune response. Here we use a model system to show that environmental cues and parasite past experience can be used by entomopathogenic nematodes to reduce individual risk of infection. Past parasite experience can more than double the infective virulence (number of host invaders) of a given cohort of entomopathogenic nematode parasites. This plasticity in individual parasite risk-taking and associated infection can be used to manage infection of parasitic nematodes: enhancing biological control with entomopathogenic nematodes and developing behavioral and chemical strategies to reduce infection by vertebrate and plant parasitic nematodes.

## Introduction

Nematode parasites—whether pathogens of vertebrates, insects, or plants—exist in dynamic systems where host infection is a critical objective [1]. While locating hosts against a backdrop of ephemeral signals is a primary challenge, nematode parasites face a secondary, and perhaps more significant challenge after arriving at their host: successful infection [1].

In a co-evolutionary arms race with nematode pathogens, their host vertebrates, insects and plants have developed a multitude of defenses to prevent exactly what the nematode parasite desires—successful infection and colonization of the host [1–3]. Indeed, this stage incurs considerable risk on the part of an individual parasitic nematode. Physical, chemical, and immune barriers all combine to prevent successful infection of the host. In vertebrate parasitic systems, Mucin and Toll receptors provide a first and second line of host defense respectively [4]. Following that gauntlet, vertebrate parasitic nematodes could then encounter a TH2 cytokine response [5, 6]. Similarly, plant parasitic nematodes encounter a multilayered defense strategy involving reactive oxygen species, production of specific secondary metabolites and fortification of cell walls [7, 8]. Insect parasitic nematodes must circumvent the insect cuticle, then will often encounter encapsulation responses [9]. Regardless of the particular defense strategy, host responses render individual infection by nematode parasites a potentially mortal risk.

Parasitic nematodes have evolved many means to address these risks in order to overcome and influence host behavior [10]. One of the most successful infection strategies involves overwhelming the host immune system through group attack [11]. This type of behavior is seen perhaps most clearly in insect parasitic nematodes, called entomopathogenic nematodes, where large numbers of pathogens are required to overcome a host’s immune system [12, 13]. Any less than the critical number and infection by too few nematode parasites results in an encapsulation response that renders a brave foray into the host an untenable and lethal outcome for the individual nematode [12, 13].

Group attack to overwhelm host responses does not necessarily obviate risk on the part of an individual entomopathogenic nematode, however. Because critical numbers are needed to achieve successful infection, any individual suffers from critically incomplete knowledge: How many parasites from their cohort will ultimately infect the same host? In a system with many decision making agents, knowledge of another’s decision can reduce an individual nematode’s risk of encapsulation [14].

Here we explore nematode parasite risk taking in the context of host infection behavior in order to understand both basic biology and the potential to manage infection in practical applications. Specifically, we address the question of whether entomopathogenic nematode infection behavior is plastic. Behavioral plasticity, learning and modifying behavior based on past experience, has been shown in relation to host-seeking and orientation behavior in entomopathogenic nematodes, but is unclear the extent to which exposure influences direct interaction with the host [15, 16]. The presence of and exposure to host plant volatiles could change entomopathogenic nematode infection behavior. Given that plant volatiles could be used to signal the presence of critical resources in the form of host availability, the hypothesis is that entomopathogenic nematodes may be more likely to infect insect hosts associated with familiar plant volatile cues.

To explore this question, we establish baseline risk profiles of infectivity, explore how environmental cues and past experience can influence that infectivity and then determine the resultant effects on host mortality. Entomopathogenic nematodes were chosen as a model study system due to their prolific nature, group dynamics, learning ability (social behavioral plasticity [15]), and use of plant volatiles as environmental cues for locating hosts [17].

## Results

### Infection

To establish baseline infectivity profiles of entomopathogenic nematodes in the presence or absence of common belowground plant volatiles, *Galleria mellonella* larvae were paired with doses of either pregeijerene or *α*-pinene then inoculated with 1000 infective juveniles of either *Heterorhabditis indica* or *Steinernema diaprepesi* in small arenas containing 1g of sterilized sand and the host insect. Pregeijerene is an herbivore-induced plant volatile known to recruit these species of entomopathogenic nematodes to *Diaprepes abbreviatus* larvae feeding on citrus roots [18, 19]. *α*-pinene is a common plant volatile recovered from the same system and is known to innately repel these species of entomopathogenic nematode [15, 18, 19]. Twenty-four and fourty-eight hours post inoculation, larval mortality and number of infective juveniles that entered the host cadaver were assessed.

Importantly, these assays were small in size. Entomopathogenic nematodes can move relatively long distances (1,000x body length) in response to volatile cues as many previous studies have established [18, 20]. Critically distinct from recruitment behavior, however, is infection of an insect host. Just because entomopathogenic nematode infective juveniles are attracted and recruited to the vicinity of the host insect does not mean the entomopathogenic nematodes will necessarily infect the insect.

**Fig 1:**
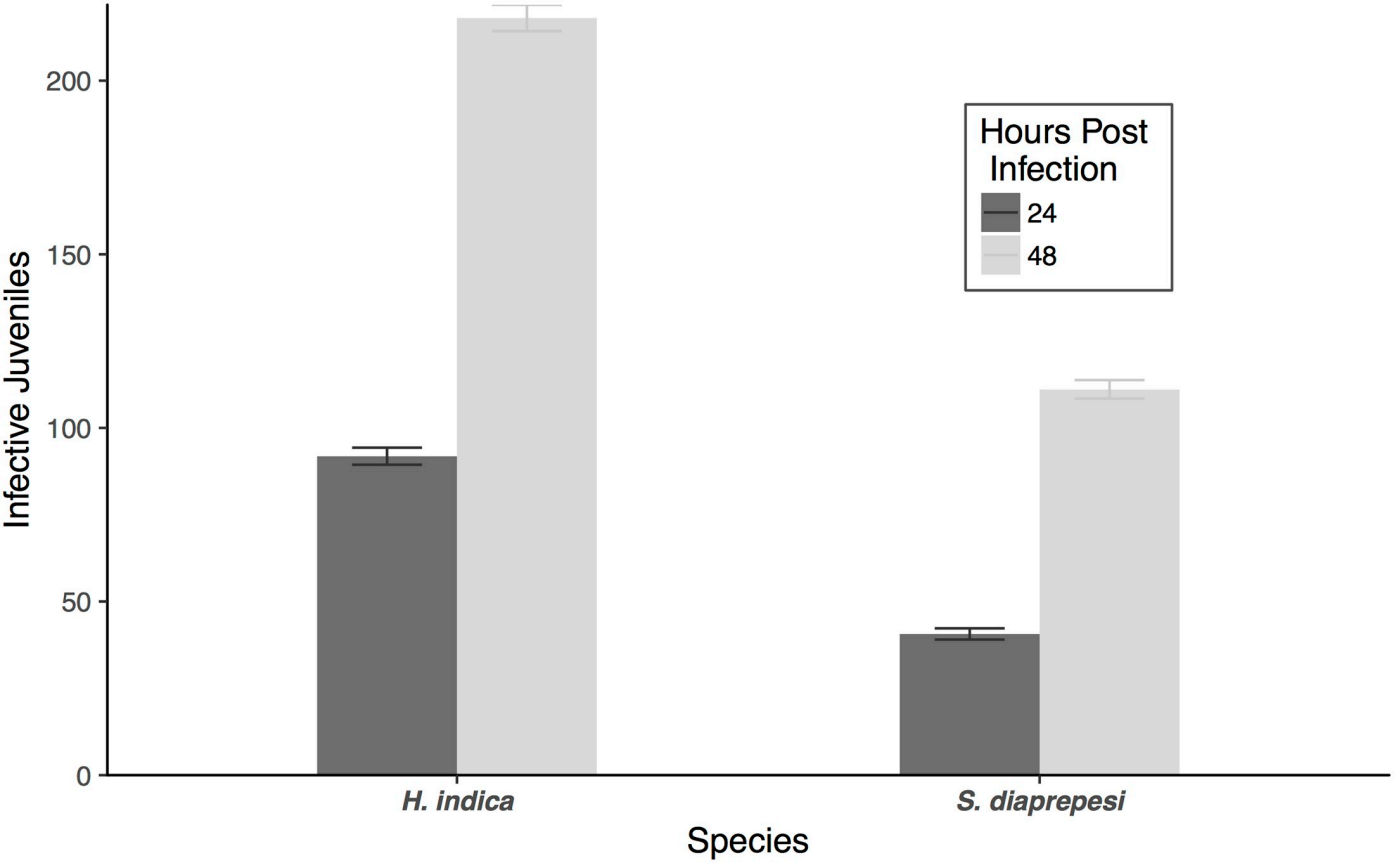
Baseline infection. Number of entomopathogenic nematode infective juveniles recovered from *G. mellonella* larvae. Bars and error bars denote means and 95% confidence intervals respectively.

In these assays that obviated long-distance recruitment, species, hours post-infection, volatile treatment, and first order interactions significantly explained observed numbers of infecting nematodes (*χ*^2^ = 325.8, 187.6, 2684.1, >628.7; *df* = 1,1,5,5; *P* < 0.001). *H. indica* infective juveniles entered the larval hosts at 2.1 [95% confidence interval: 2.04, 2.17] (Tukey HSD, *P* < 0.001) times the rate of *S. diaprepesi* infective juveniles. Additionally there were 2.37 [2.30, 2.45] and 2.73 [2.61, 2.86] (*P* < 0.001) times more infective juveniles inside the cadavers after 48 hours as compared to 24 hours for *H. indica* and *S. diaprepesi*, respectively (Fig 1).

**Fig 2:**
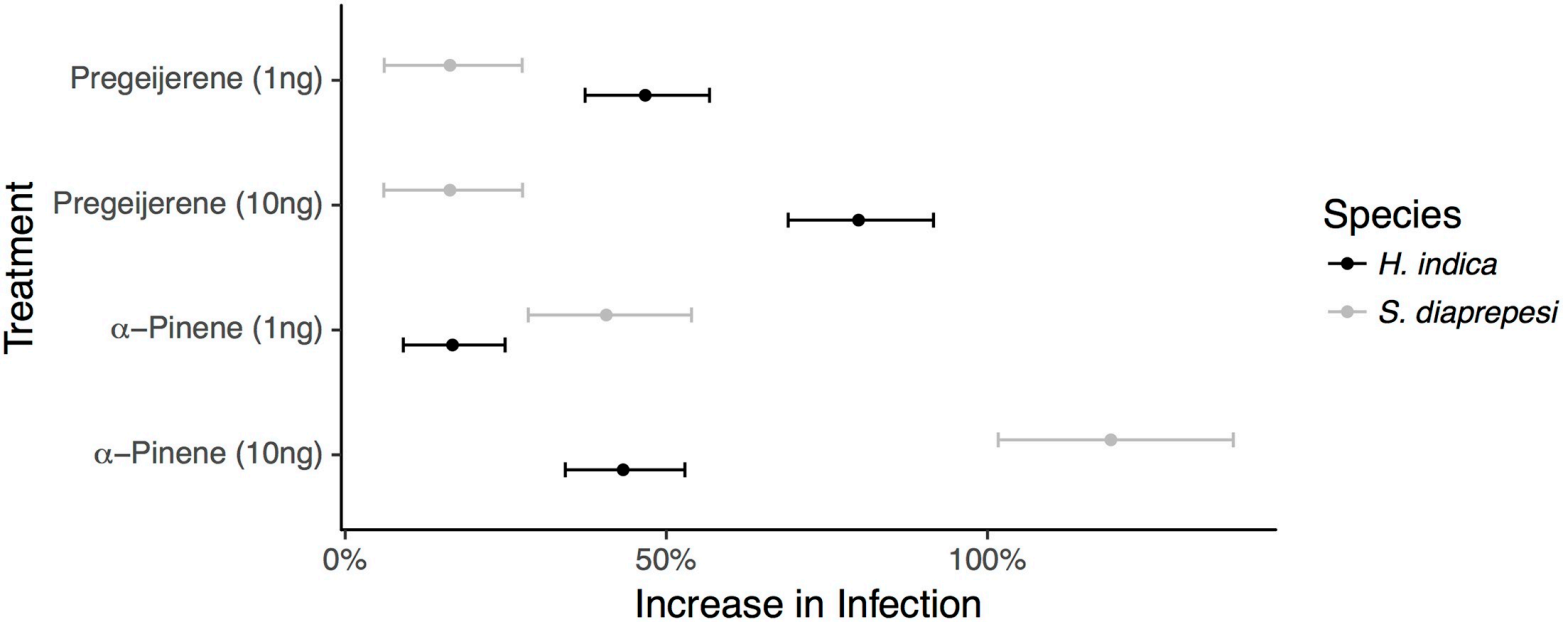
Infection increases in presence of plant volatiles. Increase in numbers of infective juveniles recovered from *G. mellonella* larvae paired with treatment volatiles. Increase is calculated relative to numbers of infective juveniles recovered from *G. mellonella* larvae not paired with treatment volatiles. Points and error bars denote means and 95% confidence intervals respectively.

Importantly, presence of any of the two plant volatiles triggered a significant (*P* < 0.001) increase in host infection by entomopathogenic nematodes over controls without plant volatiles (Fig 2, S1 Fig for raw numbers). Additionally, main effects higher doses of these volatiles resulted in higher numbers of infective juveniles recovered from cadavers for both pregeijerene and *α*-pinene (*P* < 0.001).

### Infection plasticity

To examine how past experience can change future infection behavior of parasitic nematodes, cohorts of 1000 infective juveniles were placed in glass vials containing water with or without the plant volatiles *α*-pinene or pregeijerene. Forty-eight hours later, infective juveniles were washed three times and placed into an arena containing host larvae and plant volatiles as above.

**Fig 3:**
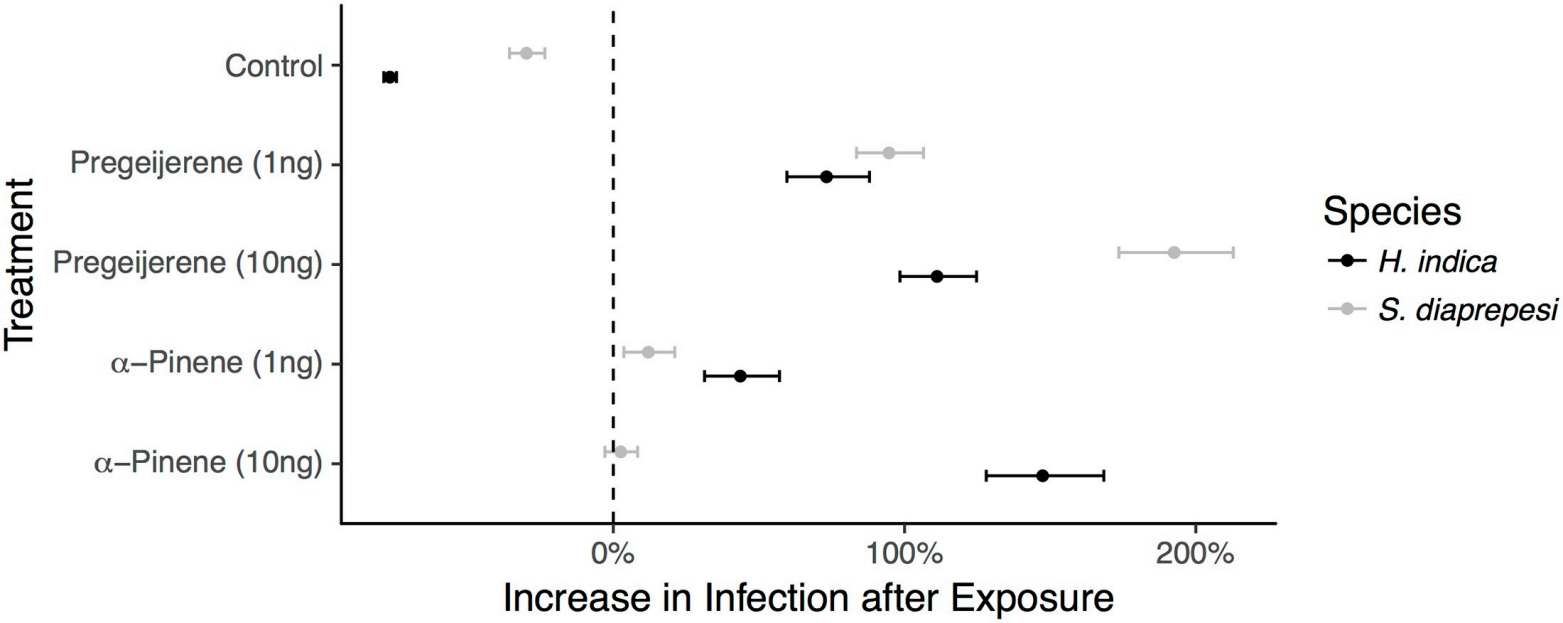
Exposure increases infection. Increase in numbers of experienced infective juveniles previously exposed to the treatment volatile recovered from *G. mellonella* larvae paired with treatment volatiles. (i.e. The entomopathogenic nematode infective juveniles infecting *G. mellonella* larvae paired with pregeijerene were previously exposed to pregeijerene.) Increase is calculated relative to numbers of inexperienced (non-exposed) infective juveniles recovered from *G. mellonella* larvae paired with treatment volatiles. Controls are nematode only controls; host insects were not treated with volatiles. Exposed cohorts in controls had experience with pregeijerene. Points and error bars denote means and 95% confidence intervals respectively.

Prior exposure to a compound in glass vials significantly increased infection (30% [27%, 33%] on average; 147% [128%, 168%] for *H. indica* exposed to 10 *ng* *α*-pinene and 192% [173%, 313%] for *S. diaprepesi* exposed to 10 *ng* pregeijerene) when that same compound was paired with the insect larva (Fig 3, S2 Fig for raw numbers). This is true for both compounds, although exposure to pregeijerene produced significantly higher increases in infection (*P* < 0.001). These effects varied by species and volatile; species, volatile treatment, exposure status, and first order interactions significantly explained observed numbers of infecting nematodes (*χ*^2^ = 68.3, 902.3, 55.2, >1081.5; *df* = 1,4,1,4; *P* < 0.001). Interestingly, prior exposure to a compound reduced infection when that compound (pregeijerene) was not paired with the host insect (60% [57%, 62%] reduction on average in nematode only controls, *P* < 0.001) (Fig 3, S2 Fig for raw numbers).

### Host mortality and susceptibility

**Fig 4:**
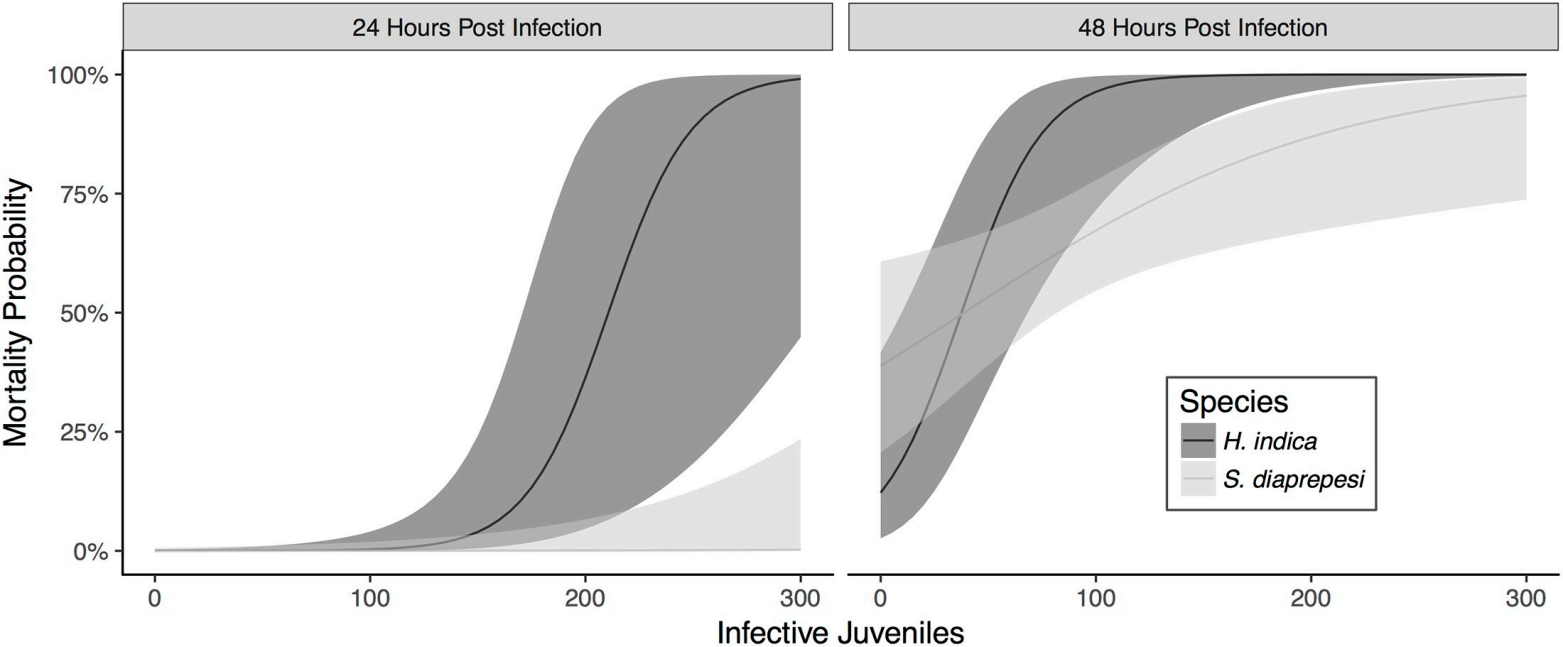
Mortality from infection. Mortality probability of *G. mellonella* larvae infected by entomopathogenic nematodes 24 and 48 hours post infection. Note that numbers of infective juveniles denote numbers infecting the host insect: those that are inside the larva. Lines and shaded regions denote estimated response and 95% confidence intervals respectively.

To determine how infection by nematode parasites influences host mortality, we used logistic regression models to estimate the probability of host death given varying levels of infection by entomopathogenic nematodes. Both the number of nematodes infecting the host insect and the time post infection had significant effects on host mortality (*χ*^2^ = 50.9, 136.9; df = 1,1; *P* < 0.001). Additionally, there was a significant interaction between species and the number of infective juveniles infecting the host (*χ*^2^ = 11.2; df = 1; *P* = 0.001). *H. indica* showed higher probabilities of host mortality for lower numbers of infecting nematodes (Fig 4). Additionally, *H. indica* infection could result in up to 100% probability of mortality in 24 hours (Fig 4). *S. diaprepesi*, in contrast, only achieved significant probability of mortality after 48 hours (Fig 4).

## Discussion

Risk taking and infection by parasitic nematodes is influenced by the host environment and past experiences of the parasitic nematode. Host insects in the presence of the plant volatiles pregeijerene and *α*-pinene can suffer an almost doubling in infection rate depending on the species assayed (Fig 2). Likewise, prior exposure to (past experience with) those same plant volatiles further increases infection, in some cases increasing infection by more than 100% (Fig 3). Interestingly, nematode parasites previously exposed to a volatile are less likely to infect a host insect if the volatile to which they had been exposed is not present. This observation corroborates previous observations that volatile exposure confers specific preference [15]; nematode parasites prefer volatiles with which they have experience, but seem to lose interest in the absence of that volatile.

That presence of and prior exposure to plant volatiles alters infection profiles of entomopathogenic nematodes suggests that these volatiles could perhaps be functioning as cues to answer that critical question: How many compatriot parasites will ultimately infect the same host? Such volatiles could be used as a proxy for infection potential. Evolutionarily, if many nematode parasites rely on similar environmental cues, any individual parasite is more likely to successfully infect a host if one of those similar environmental cues is present. The risk to any individual is reduced.

The relationship between individual parasite risk and reward is perhaps seen most clearly in examining the probability of host mortality as a function of number of infecting nematodes (Fig 4). For these insect parasitic nematodes, an appreciable probability of host mortality (>75%) is achieved at somewhere between 50-100+ infecting nematodes after two days. Importantly, it is worth noting that the insect species being infected—larvae of the greater wax moth (*Galleria mellonella*)—is known for and used in entomopathogenic nematode rearing because of its relatively weak and maladapted immune system [21]. In other, adapted, hosts, high probability of insect host mortality would likely necessitate an even greater number of parasites entering the host to facilitate successful infection.

Because successful infection is the primary objective of parasitic nematodes upon locating a host, it is interesting that environmental cues and learned experience can augment infection potential. Presence of and past exposure to plant volatiles can increase entomopathogenic nematode virulence. Enhanced virulence is plastic and can be learned based on past experience.

This plasticity can influence the lifecycle of nematodes in the natural environment and be appropriated for biological control of insect pests. Entomopathogenic nematodes emerging from their host cadaver likely are exposed to plant volatiles that may influence infection potential in similar situations in the future. Additionally, by isolating infection behavior in assays obviating long-distance recruitment, our results inform previous observations that recruitment alone is not sufficient to account for increased host insect mortality in comparatively large olfactometers [16]. This pattern of enhanced infection following volatile exposure can also inform biological control solutions where entomopathogenic nematodes can be exposed to volatile cues *in-vitro* in order to enhance their infection in agricultural fields.

The ability of nematode parasites to demonstrate infection plasticity based on environmental cues and past experience is not limited to management of entomopathogenic nematodes, however. Many nematode parasites of vertebrates, plants and insects demonstrate similar lifecycles and share common adaptations. Animal and plant nematode parasites, for example, respond strongly to host associated plant volatiles [22] and many share conserved pheromone signaling mechanisms [23]. Management and knowledge of environmental cues and past experience will likely aid in managing nematode parasites in a variety of settings: enhancing biological control with entomopathogenic nematodes and reducing infection of vertebrate and plant parasitic nematodes are current examples of potential practical application.

## Materials and methods

### Organisms

Entomopathogenic nematode infective juveniles of *Heterorhabditis indica* and *Steinernema diaprepes* were originally isolated from Florida citrus groves (near Homestead and Bartow Florida respectively) then cultivated in *Galleria mellonella* waxworm larvae until use in experiments [24]. This study was not a field trial and only involved laboratory work. Strains used in this study were originally isolated from natural populations in Florida at locations used by the University of Florida. Permissions for nematode collection are not required at those locations. The strains isolated from those locations and used in this study are standard laboratory strains used in a multitude of laboratory trials and similarly used in many of the references cited in this manuscript. Upon emerging from larvae, infective juveniles were collected in White traps then used within a week in bioassays [25].

### Bioassays

Bioassay arenas consisted of one gram of clean autoclaved sand adjusted to 10% moisture by volume placed into each well of a 24 well plate (1.56cm bottom diameter). One host *G. mellonella* larva was added to each well along with respective volatile treatment. Following arena setup and application of volatile treatment, 1000 entomopathogenic nematode infective juveniles were added to each well. Either 24 or 48 hours later, mortality of *G. mellonella* was determined by observing responses to prodding with a needle. Following mortality determination, larvae were frozen. After freezing, larvae underwent pepsin digestion and infective juveniles that had entered the larvae were extracted and counted as previously described [26, 27].

### Experimental design

To determine how the presence of plant volatiles influences entomopathogenic nematode infection, sand arenas with *G. mellonella* larvae were inoculated with 10 *μl* of pentane containing either 10 *ng* of pregeijerene (extracted from the roots of the Common Rue plant *Ruta graveolens*), 1 *ng* pregeijerene, 10 *ng* *α*-pinene (Sigma Aldrich, 98%), or 1 *ng* *α*-pinene. Twelve replications of each treatment were conducted.

To determine how prior exposure to plant volatiles affects entomopathogenic nematode infectivity, cohorts of 1000 infective juveniles were placed in vials containing 10ml DI water and 5 *μl* of either 10 *ng*/*μl* pregeijerene or 10 *ng*/*μl* *α*-pinene in pentane. Vials containing these exposed cohorts were incubated at 25°C for 48 hours before washing the infective juveniles three times. For comparison, non-exposed cohorts were also established as above with the exception that 5 *μl* pentane was used in place of volatile treatment. These cohorts were then placed into arenas constructed as above and treated with either 10 *ng* of pregeijerene, 1 *ng* pregeijerene, 10 *ng* *α*-pinene, 1 *ng* *α*-pinene, or pentane such that volatile treatments matched pre-exposure. As above, twelve replications of each treatment were conducted.

Additionally, each of the trials above had three different controls each consisting of twelve replications. The first control consisted of host insects prepared as above to which no nematodes were added. This control was included to check for possible contamination by rogue entomopathogenic nematodes that could have (however unlikely) escaped culture. In all cases, these controls returned negative results; no nematodes were ever recovered and no host insects suffered mortality. The second control consisted of nematodes only where the host insect was prepared as above with the exception that no plant volatiles were included in the arena. This control was designed to establish a baseline level of infection in the absence of host environmental cues. The third control consisted of host insects treated with pentane only. This control was designed to verify that the small amount of pentane solvent was not altering entomopathogenic nematode response. Because of the results of these controls, it is highly unlikely that pentane increases entomopathogenic nematode response. Pentane only controls resulted in either slightly less infection than (*P* < 0.0001) or were not significantly different from no pentane controls (*P* = 0.08).

### Analysis

Entomopathogenic nematode infectivity was evaluated with Poisson regression. Species, treatment, hours post infection, replication number, exposure status and two factor interactions were considered for their ability to explain observed numbers of infective juveniles in the insect cadaver. Models were selected based on analysis of deviance, information criteria, goodness of fit metrics, psuedo *R*^2^ values, and residual diagnostics.

The effects and interactions between number of infective juveniles entering a cadaver, hours post infection, treatment, and nematode species on *G. mellonella* mortality was evaluated using logistic regression. Best fit models were chosen based on analysis of deviance, information criteria, goodness of fit metrics, psuedo *R*^2^ values, and residual diagnostics.

Data were collated in Microsoft Excel then imported into R version 3.3.1 [28] for analysis in the RStudio version 1.0.136 development environment [29]. The following packages facilitated analysis: *readxl* for the R-Excel interface [30], *tidyverse* for creating a tidy data environment [31], *car* for analysis of deviance [32], and *lsmeans* for post-hoc comparisons [33].

## Supporting information

S1 Fig Baseline infection with volatile treatments. Points and error bars denote mean number of nematodes recovered from the host insect cadaver and 95% confidence intervals respectively. Controls are nematode only controls; host insects were not treated with volatiles.
(TIFF)

S2 Fig Infection following prior exposure to volatile treatments. Points and error bars denote mean number of nematodes recovered from the host insect cadaver and 95% confidence intervals respectively. Controls are nematode only controls; host insects were not treated with volatiles. Exposed cohorts had experience with pregeijerene.
(TIFF)
