# Supplementary figures and images for: Risk taking of educated nematodes
Source: PLoS One. 2018 Oct 25;13(10):e0205804. doi: 10.1371/journal.pone.0205804 (PMC6201888; doi:10.1371/journal.pone.0205804)

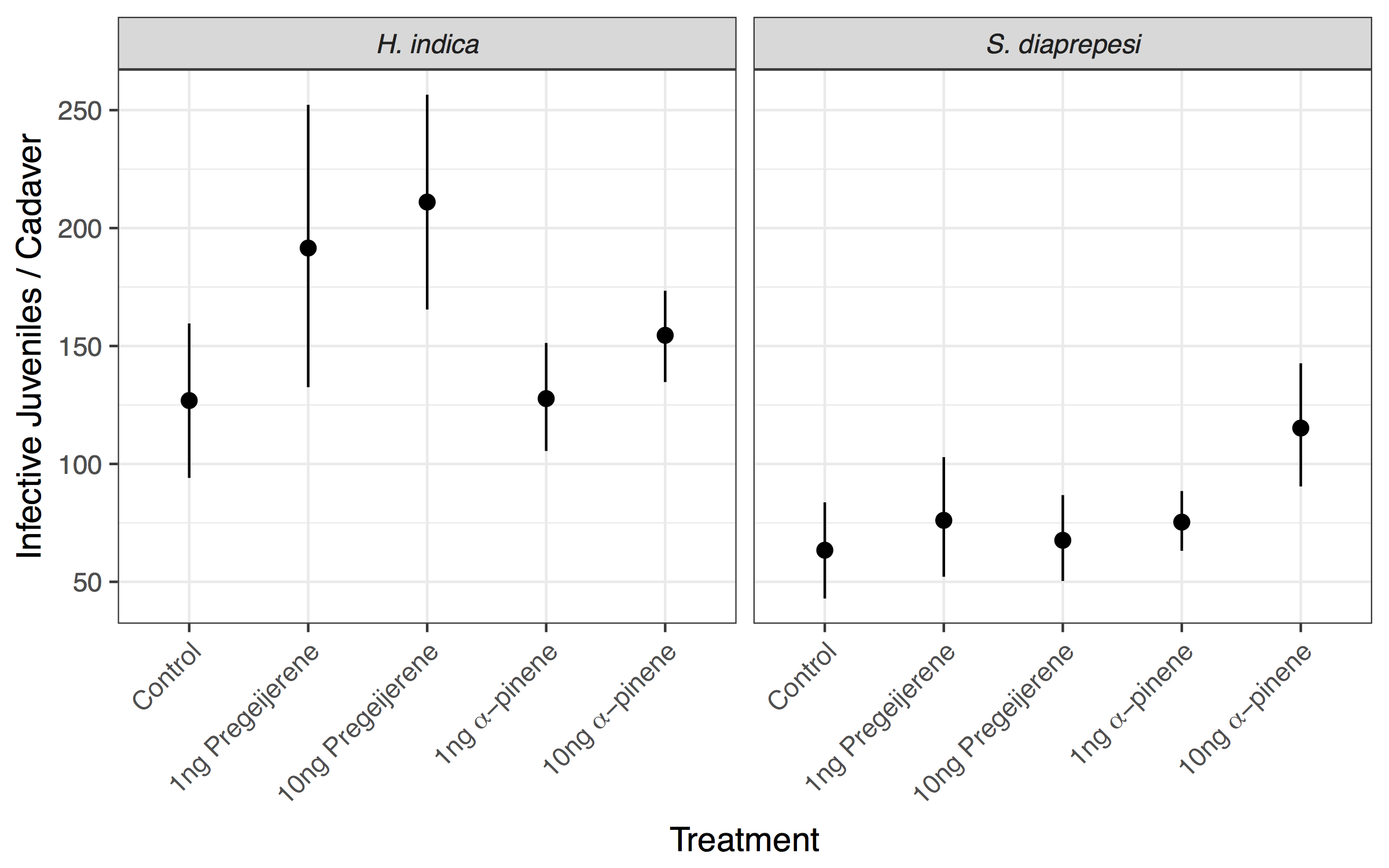

Supplement: S1 Fig — Points and error bars denote mean number of nematodes recovered from the host insect cadaver and 95% confidence intervals respectively. Controls are nematode only controls; host insects were not treated with volatiles. (TIFF) [file pone.0205804.s001.tiff]

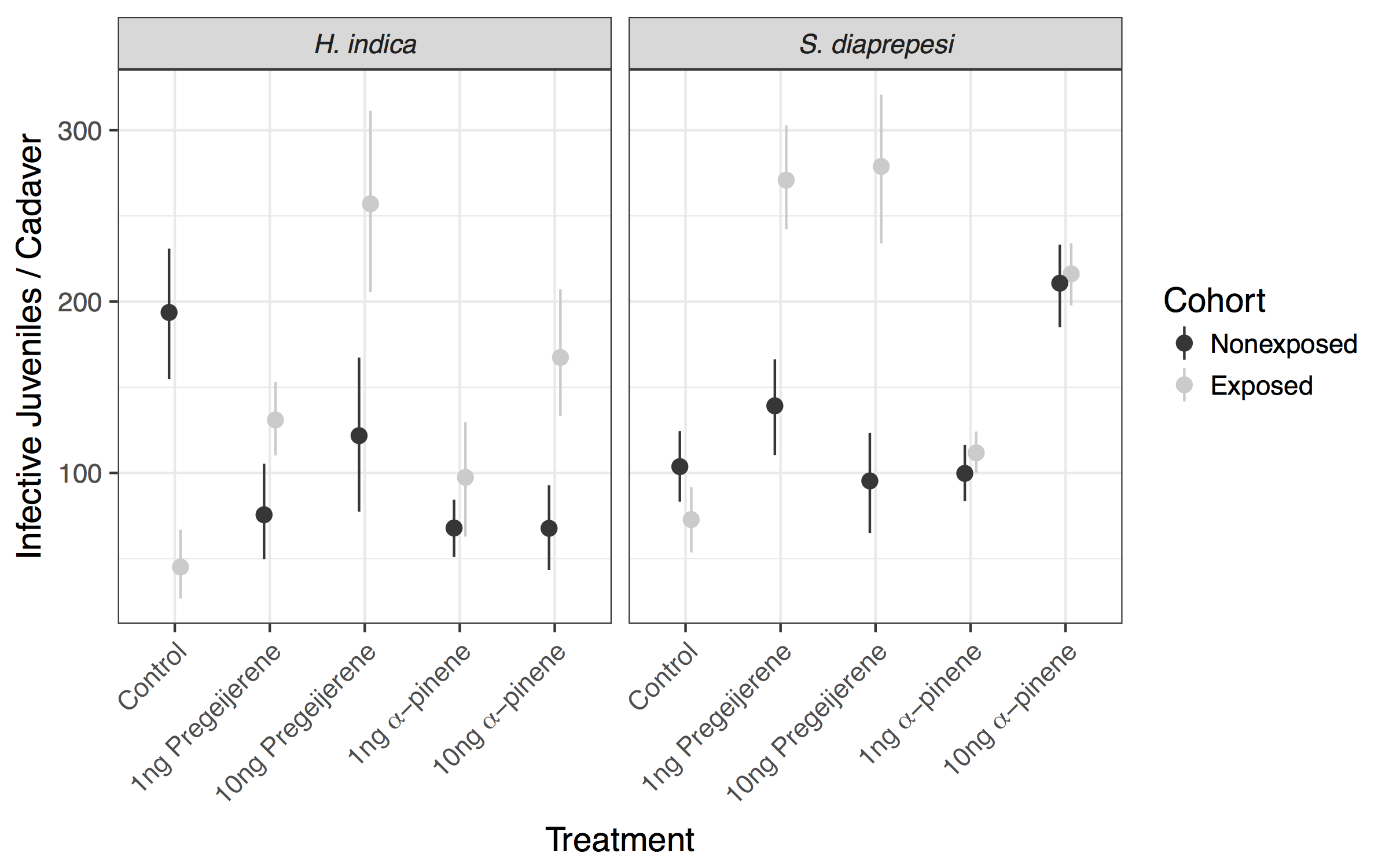

Supplement: S2 Fig — Points and error bars denote mean number of nematodes recovered from the host insect cadaver and 95% confidence intervals respectively. Controls are nematode only controls; host insects were not treated with volatiles. Exposed cohorts had experience with pregeijerene. (TIFF) [file pone.0205804.s002.tiff]
